# Effects of music therapy on depression: A meta-analysis of randomized controlled trials

**DOI:** 10.1371/journal.pone.0240862

**Published:** 2020-11-18

**Authors:** Qishou Tang, Zhaohui Huang, Huan Zhou, Peijie Ye

**Affiliations:** 1 Bengbu Medical University, Bengbu, Anhui, China; 2 Anhui Provincial Center for Women and Child Health, Hefei, Anhui, China; 3 National Drug Clinical Trial Institution, The First Affiliated Hospital of Bengbu Medical University, Bengbu, Anhui, China; Anadolu University, TURKEY

## Abstract

**Background:**

We aimed to determine and compare the effects of music therapy and music medicine on depression, and explore the potential factors associated with the effect.

**Methods:**

PubMed (MEDLINE), Ovid-Embase, the Cochrane Central Register of Controlled Trials, EMBASE, Web of Science, and Clinical Evidence were searched to identify studies evaluating the effectiveness of music-based intervention on depression from inception to May 2020. Standardized mean differences (SMDs) were estimated with random-effect model and fixed-effect model.

**Results:**

A total of 55 RCTs were included in our meta-analysis. Music therapy exhibited a significant reduction in depressive symptom (SMD = −0.66; 95% CI = -0.86 to -0.46; *P*<0.001) compared with the control group; while, music medicine exhibited a stronger effect in reducing depressive symptom (SMD = −1.33; 95% CI = -1.96 to -0.70; *P*<0.001). Among the specific music therapy methods, recreative music therapy (SMD = -1.41; 95% CI = -2.63 to -0.20; *P*<0.001), guided imagery and music (SMD = -1.08; 95% CI = -1.72 to -0.43; *P*<0.001), music-assisted relaxation (SMD = -0.81; 95% CI = -1.24 to -0.38; *P*<0.001), music and imagery (SMD = -0.38; 95% CI = -0.81 to 0.06; *P* = 0.312), improvisational music therapy (SMD = -0.27; 95% CI = -0.49 to -0.05; *P* = 0.001), music and discuss (SMD = -0.26; 95% CI = -1.12 to 0.60; *P* = 0.225) exhibited a different effect respectively. Music therapy and music medicine both exhibited a stronger effects of short and medium length compared with long intervention periods.

**Conclusions:**

A different effect of music therapy and music medicine on depression was observed in our present meta-analysis, and the effect might be affected by the therapy process.

## Introduction

Depression was reported to be a common mental disorders and affected more than 300 million people worldwide, and long-lasting depression with moderate or severe intensity may result in serious health problems [1]. Depression has become the leading causes of disability worldwide according to the recent World Health Organization (WHO) report. Even worse, depression was closely associated with suicide and became the second leading cause of death, and nearly 800 000 die of depression every year worldwide [1, 2]. Although it is known that treatments for depression, more than 3/4 of people in low and middle-income income countries receive no treatment due to a lack of medical resources and the social stigma of mental disorders [3]. Considering the continuously increased disease burden of depression, a convenient effective therapeutic measures was needed at community level.

Music-based interventions is an important nonpharmacological intervention used in the treatment of psychiatric and behavioral disorders, and the obvious curative effect on depression has been observed. Prior meta-analyses have reported an obvious effect of music therapy on improving depression [4, 5]. Today, it is widely accepted that the music-based interventions are divided into two major categories, namely music therapy and music medicine. According to the American Music Therapy Association (AMTA), “music therapy is the clinical and evidence-based use of music interventions to accomplish individualized goals within a therapeutic relationship by a credentialed professional who has completed an approved music therapy program” [6]. Therefore, music therapy is an established health profession in which music is used within a therapeutic relationship to address physical, emotional, cognitive, and social needs of individuals, and includes the triad of music, clients and qualified music therapists. While, music medicine is defined as mainly listening to prerecorded music provided by medical personnel or rarely listening to live music. In other words, music medicine aims to use music like medicines. It is often managed by a medical professional other than a music therapist, and it doesn’t need a therapeutic relationship with the patients. Therefore, the essential difference between music therapy and music medicine is about whether a therapeutic relationship is developed between a trained music therapist and the client [7–9]. In the context of the clear distinction between these two major categories, it is clear that to evaluate the effects of music therapy and other music-based intervention studies on depression can be misleading. While, the distinction was not always clear in most of prior papers, and no meta-analysis comparing the effects of music therapy and music medicine was conducted. Just a few studies made a comparison of music-based interventions on psychological outcomes between music therapy and music medicine. We aimed to (1) compare the effect between music therapy and music medicine on depression; (2) compare the effect between different specific methods used in music therapy; (3) compare the effect of music-based interventions on depression among different population [7, 8].

## Materials and methods

### Search strategy and selection criteria

PubMed (MEDLINE), Ovid-Embase, the Cochrane Central Register of Controlled Trials, EMBASE, Web of Science, and Clinical Evidence were searched to identify studies assessing the effectiveness of music therapy on depression from inception to May 2020. The combination of “depress*” and “music*” was used to search potential papers from these databases. Besides searching for electronic databases, we also searched potential papers from the reference lists of included papers, relevant reviews, and previous meta-analyses. The criteria for selecting the papers were as follows:(1) randomised or quasi-randomised controlled trials; (2) music therapy at a hospital or community, whereas the control group not receiving any type of music therapy; (3) depression rating scale was used. The exclusive criteria were as follows: (1) non-human studies; (2) studies with a very small sample size (n<20); (3) studies not providing usable data (including sample size, mean, standard deviation, etc.); (4) reviews, letters, protocols, etc. Two authors independently (YPJ, HZH) searched and screened the relevant papers. EndNote X7 software was utilized to delete the duplicates. The titles and abstracts of all searched papers were checked for eligibility. The relevant papers were selected, and then the full-text papers were subsequently assessed by the same two authors. In the last, a panel meeting was convened for resolving the disagreements about the inclusion of the papers.

### Data extraction

We developed a data abstraction form to extract the useful data: (1) the characteristics of papers (authors, publish year, country); (2) the characteristics of participators (sample size, mean age, sex ratio, pre-treatment diagnosis, study period); (3) study design (random allocation, allocation concealment, masking, selection process of participators, loss to follow-up); (4) music therapy process (music therapy method, music therapy period, music therapy frequency, minutes per session, and the treatment measures in the control group); (5) outcome measures (depression score). Two authors independently (TQS, ZH) abstracted the data, and disagreements were resolved by discussing with the third author (YPJ).

### Assessment of risk of bias in included studies

Two authors independently (TQS, ZH) assessed the risk of bias of included studies using Cochrane Collaboration’s risk of bias assessment tool, and disagreements were resolved by discussing with the third author (YPJ) [10].

### Music therapy and music medicine

Music Therapy is defined as the clinical and evidence-based use of music interventions to accomplish individualized goals within a therapeutic relationship by a credentialed professional who has completed an approved music therapy program. Music medicine is defined as mainly listening to prerecorded music provided by medical personnel or rarely listening to live music. In other words, music medicine aims to use music like medicines.

Music therapy mainly divided into active music therapy and receptive music therapy. Active music therapy, including improvisational, re-creative, and compositional, is defined as playing musical instruments, singing, improvisation, and lyrics of adaptation. Receptive music therapy, including music-assisted relaxation, music and imagery, guided imagery and music, lyrics analysis, and so on, is defined as music listening, lyrics analysis, and drawing with musing. In other words, in active methods participants are making music, and in receptive music therapy participants are receiving music [6, 7, 9, 11–13].

### Evaluation of depression

Depression was evaluated by the common psychological scales, including Beck Depression Inventory (BDI), Children’s Depression Inventory (CDI), Center for Epidemiologic Studies Depression (CES-D), Cornell Scale (CS), Depression Mood Self-Report Inventory for Adolescence (DMSRIA), Geriatric Depression Scale-15 (GDS-15); Geriatric Depression Scale-30 (GDS-30), Hospital Anxiety and Depression Scale (HADS), Hamilton Rating Scale for Depression (HRSD/HAMD), Montgomery-sberg Depression Rating Scale (MADRS), Patient Reported Outcomes Measurement Information System (PROMIS), Self-Rating Depression Scale (SDS), Short Version of Profile of Mood States (SV-POMS).

### Statistical analysis

The pooled effect were estimated by using the standardized mean differences (SMDs) and its 95% confidence interval (95% CI) due to the different depression rate scales were used in the included papers. Heterogeneity between studies was assessed by I-square (*I*^*2*^) and Q-statistic (P<0.10), and a high *I*^*2*^ (>50%) was recognized as heterogeneity and a random-effect model was used [14–16]. We performed subgroup analyses and meta-regression analyses to study the potential heterogeneity between studies. The subgroup variables included music intervention categories (music therapy and music medicine), music therapy methods (active music therapy, receptive music therapy), specific receptive music therapy methods (music-assisted relaxation, music and imagery, and guided imagery and music (Bonny Method), specific active music therapy methods (recreative music therapy and improvisational music therapy), music therapy mode (group therapy, individual therapy), music therapy period (weeks) (2–4, 5–12, ≥13), music therapy frequency (once weekly, twice weekly, ≥3 times weekly), total music therapy sessions (1–4, 5–8, 9–12, 13–16, >16), time per session (minutes) (15–40, 41–60, >60), inpatient settings (secure [locked] unit at a mental health facility versus outpatient settings), sample size (20–50, ≥50 and <100, ≥100), female predominance(>80%) (no, yes), mean age (years) (<50, 50–65, >65), country having music therapy profession (no, yes), pre-treatment diagnosis (mental health, depression, severe mental disease/psychiatric disorder). We also performed sensitivity analyses to test the robustness of the results by re-estimating the pooled effects using fixed effect model, using trim and fill analysis, excluding the paper without information on music therapy, excluding the papers with more high biases, excluding the papers with small sample size (20< n<30), excluding the papers using an infrequently used scale, excluding the studies focused on the people with a severe mental disease. We investigated the publication biases by a funnel plot as well as Egger’s linear regression test [17]. The analyses were performed using Stata, version 11.0. All P-values were two-sided. A P-value of less than 0.05 was considered to be statistically significant.

## Results

### Characteristics of the eligible studies

**Fig 1:**
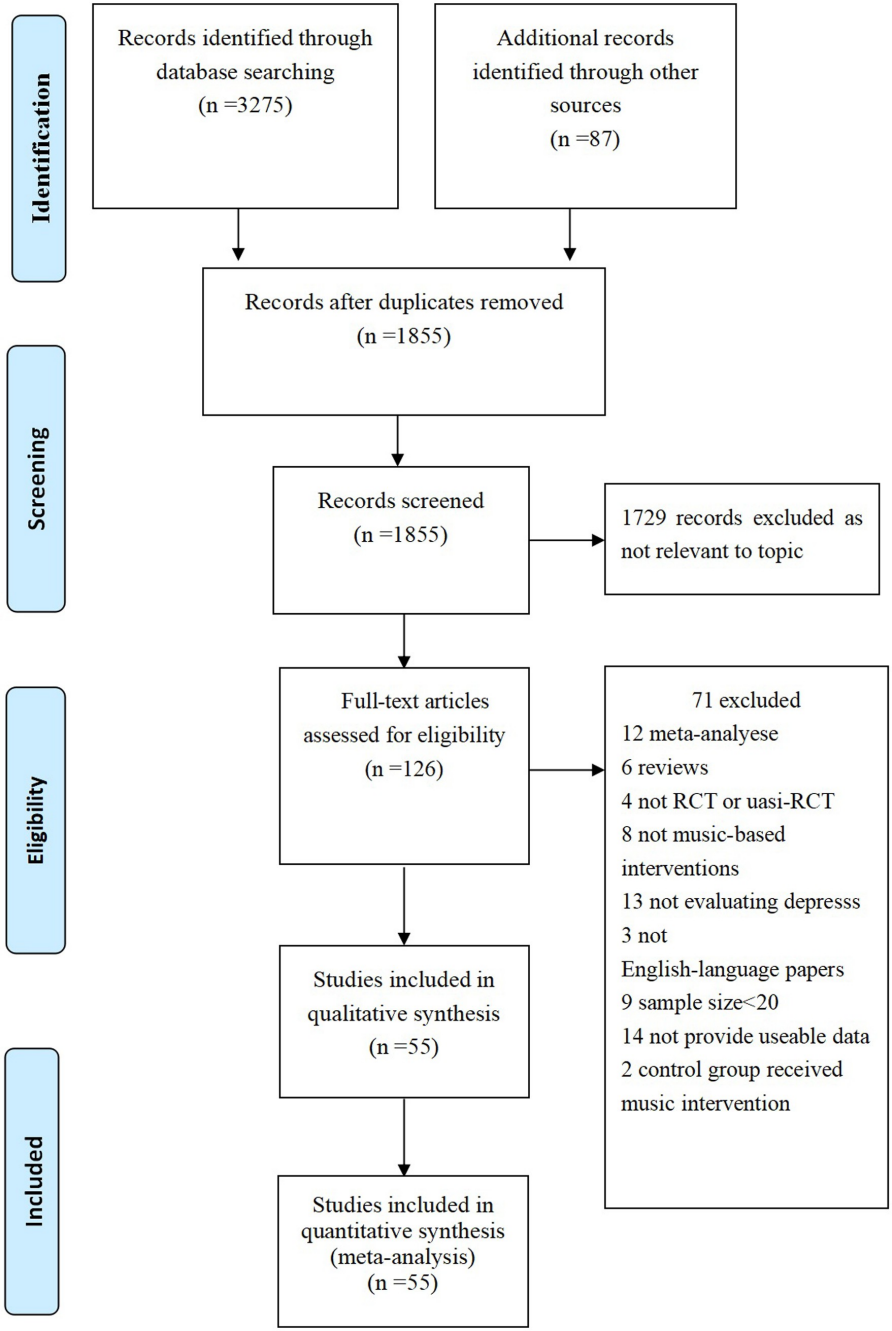
Prisma 2009 flow diagram literature search and study selection. PRISMA diagram showing the different steps of systematic review, starting from literature search to study selection and exclusion. At each step, the reasons for exclusion are indicated. Doi: 10.1371/journal.pone.0052562.g001.

Fig 1 depicts the study profile, and a total of 55 RCTs were included in our meta-analysis [18–72]. Of the 55 studies, 10 studies from America, 22 studies from Europe, 22 studies from Asia, and 1 study from Australia. The mean age of the participators ranged from 12 to 86; the sample size ranged from 20 to 242. A total of 16 different scales were used to evaluate the depression level of the participators. A total of 25 studies were conducted in impatient setting and 28 studies were in outpatients setting; 32 used a certified music therapist, 15 not used a certified music therapist (for example researcher, nurse), and 10 not reported relevent information. A total of 16 different depression rating scales were used in the included studies, and HADS, GDS, and BDI were the most frequently used scales (Table 1).

**Table 1:** Characteristics of clinical trials included in this meta-analysis.

Studies	Country	Ample size	Mean age (SD)	Pre-intervention diagnosis	Music intervention method (total)	Intervenor or therapist	Intervention description	Control group	Outcome Measures
Biasutti et al., 2019	Italy	N = 45, Female = 29	84.6 (7.17)	Healthy or with cognitive impairment	Active music therapy (improvisational music therapy)	Certified music therapist	Twice weekly (70 min/session) for 6 weeks	45-minute gymnastic activities	GDS-15
Burrai et al., [48]	Italy	N = 159, Female = 124	73.05 (11.5)	Heart failure	Music medicine	Researchers	Once daily (30 min/session) for 36 weeks	Standard HF treatment	HADS
Burrai et al., [49]	Italy	N = 24, Female = 9	62.3(2.8)	End-stage kidney disease	Music medicine	Nurse	Once daily (15 min/session) for 2 weeks	Standard hemodialysis	HADS
Chan et al., 2009	Hong Kong China	N = 47, Female = 26	>60	No mental illness	Music medicine	Researchers	Once weekly (30 min/session) for 4 weeks	Without any intervention	GDS-30
Chan et al., 2010	Hong Kong China	N = 42, Female = 23	>60	No mental illness	Music medicine	Researchers	Once weekly (45 min/session) for 4 weeks	Without any intervention	GDS-15
Chan et al., 2012	Singapore	N = 50, Female = 32	>55	No mental illness	Music medicine	Researchers	Once weekly (30 min/session) for 8 weeks	Without any intervention	GDS-15
Chen et al., 2015	Taiwan China	N = 71, Female = 69	18.5	Depressive disorder	Music medicine	Researchers	Twice weekly (40 min/session) for 10 weeks	Without any intervention	DMSRIA
Chen et al., 2018	China	N = 52, Female = 52	-	Breast cancer	Receptive music therapy	Certified music therapist	Once weekly (60 min/session) for 8 weeks	Standard care	HADS
Chen et al., 2019	Taiwan China	N = 65, Female = 56	72.7(5.97)	No mental illness	Active music therapy (improvisational music therapy)	Not reported	Twice weekly (40 min/session) for 10 weeks	No music therapy	BDI
Cheung et al., 2019	Hong Kong, China	N = 60, Female = 25	13.2(3.27)	Pediatric brain tumor with a significant level of depression	Active music therapy (recreative music therapy)	Certified music therapist	Once weekly (45 min/session) for 52 weeks	No music therapy	CES-D
Chirico et al., 2020	Italy	N = 64, Female = 64	55.95(5.92)	Breast cancer	Receptive music therapy	Certified music therapist	20 min/session	Standard care	SV-POMS
Choi et al., 2008	Korea	N = 26, Female = 14	36.15(10.2)	Psychiatric disorder	Active music therapy (recreative music therapy)	Certified music therapist	Once-two weekly (60 min/session) for 12 weeks	Routine care	BDI
Chu et al., 2014	Taiwan, China	N = 100, Female = 53	82(6.8)	Dementia	Active music therapy (improvisational music therapy)	Certified music therapist	Twice weekly (30 min/session) for 6 weeks	Standard care	CS
Cooke et al., 2010	Australia	N = 47, Female = 33	>65	Dementia	Active music therapy (improvisational music therapy)	Musicians	Thrice weekly (40 min/session) for 8 weeks	Educational/entertainment activities	GDS
Erkkilä et al., 2011	Finland	N = 79, Female = 62	35.6(9.75)	Depression disorder	Active music therapy (improvisational music therapy)	Certified music therapist	Twice weekly (60 min/session) for 12 weeks	Standard treatment	MADRS
Fancourt et al., 2019	UK	N = 62, Female = 48	54.5 (14.5)	Cancer carers	Active music therapy (improvisational music therapy)	Certified music therapist	Once weekly (90 min/session) for 12 weeks	No music therapy	HADS
Gok Ugur et al., 2017	Turkey	N = 64, Female = 22	76.35(7.88)	No mental illness	Receptive music therapy (music and imagery)	Certified music therapist	Three days in a week for 8 weeks	No music therapy	GDS-15
Guétin et al., 2009	France	N = 30, Female = 22	86(5.6)	Moderate stages of Alzheimer’s disease	Receptive music therapy (music-assisted relaxation)	Certified music therapist	Once weekly (20 min/session) for 16 weeks	Educational/entertainment activities	GDS-30
Hanser et al., 1994	USA	N = 30, Female = 23	67.9	Depressive disorder	Receptive music therapy (guided imagery and music)	Certified music therapist	Once weekly (1 h/session; 20 min/session) for 8 weeks	No music therapy	GDS
Hars et al., 2014	Switzerland	N = 134, Female = 129	75(7)	No mental illness	Music medicine	Not reported	Once weekly (1 h/session) for 26 weeks	No music therapy	HADS
Liao et al., 2018	China	N = 107, Female = 66	71.79(7.71)	Mild to moderate depressive symptoms	Music medicine	Not reported	Once weekly (50 min/session) for 12 weeks	Routine health education	GDS-30
Low et al., 2020	USA	N = 43, Female = 33	50.07(5.48)	Chronic pain	Active+receptive music therapy	Certified music therapist	Once weekly (90 min/session) for 12 weeks	Standard care	PROMIS
Mahendran et al., 2018	Singapore	N = 68, Female = 56	71.1(5.3)	Mild cognitive impairment	Receptive music therapy (guided imagery and music)	Certified music therapist	Once weekly for 3 months, then fortnightly for 36 weeks.	No music therapy	GDS-15
Park et al., 2015	South Korea	N = 29, Female = 16	8.17(1.47)	No mental illness	Active music therapy (improvisational music therapy)	Music therapist	Once weekly (120 min/session) for 15 weeks	Educational creative movement program	CDI
Pérez-Ros et al., 2019	Spain	N = 119, Female = 61	80.52(7.44)	No mental illness	Active music therapy (improvisational music therapy)	Physiotherapists	5 times weekly (60 min/session) for 8 weeks	No music therapy	CS
Ploukou et al., 2018	Greece	N = 48, Female = 46	-	Oncology nurses without diseases	Music medicine	Not reported	Once weekly (60 min/session) for 4 weeks	No music therapy	HADS
Ribeiro et al., 2018	Brazil	N = 21, Female = 21	22.5(6.5)	Mothers of preterm	Receptive music therapy (music and discuss)	Certified music therapist	Once weekly (30–40 min/session) for 7–9 weeks	No music therapy	BDI
Sigurdardóttir et al., 2019	Denmark	N = 38, Female = 25	25.4	Mild and moderate depression	Music medicine	Not reported	Twice weekly (20 min/session) for 4 weeks	No music therapy	HRSD-6, HRSD-17
Toccafondi et al., 2018	Italy	N = 242, Female = 147	>18	Cancer	Receptive music therapy	Certified music therapist	Once weekly	Standard care	HADS
Trimmer et al., 2018	Canada	N = 28, Female = 15	43(13.8)	Depression and anxiety	Active music therapy (recreative music therapy)	Not reported	Once weekly (90 min/session) for 9 weeks	Treatment as usual	HADS
Volpe et al., 2018	Italy	N = 106, Female = 106	43.83(12.7)	Psychosis	Active music therapy (improvisational music therapy)	Certified music therapist	Twice daily (60 min/session) for 6 weeks	Standard drug treatment	HADS
Wu et al., 2019	China	N = 60, Female = 60	36.2(9.47)	Methamphetamine use disorder	Active+receptive music therapy	Certified music therapist	Once weekly (90 min/session) for 13 weeks	Standard treatment	SDS
Albornoz et al., 2011	Venezuela	N = 24, Female = 0	16–60	Depressed adults with substance abuse	Active music therapy (improvisational music therapy)	Therapist	Once weekly (120 min/session) for 12 weeks	Standard treatment	BDI, HRSD
Hendricks et al., 1999	USA	N = 20	14–15	Depression	Active+receptive music therapy	Therapist	Once weekly for 8 weeks	Individual psychotherapy	BDI
Hendricks et al., 2001	USA	N = 63	12–18	Depression	Music medicine	counsellor-researcher	Once weekly (60 min/session) for 12 weeks	Cognitive-based psychotherapy	BDI
Radulovic et al., 1996	Serbia	N = 60	21–62 (40)	Depression	Receptive music therapy	Therapist	Twice weekly (20 min/session) for 6 weeks	Treatment as usual	BDI
Zerhusen et al., 1995	USA	N = 60	70–82 (77)	Moderate to severe depression	Music medicine	Not reported	Twice weekly (30 min/session) for 10 weeks	psychological therapy or treatment as usual	BDI
Chang et al., 2008	Taiwan China	N = 236, Female = 236	22-41(30.03)	Pregnant women	Music medicine	Music faculty members	Once a day (30 min/session) for 2 weeks	General prenatal care	EPDS
Chen et al., 2020	Taiwan China	N = 100 Female = 100	30.19(9.50)	Beast cancer undergoing chemotherapy.	Receptive music therapy	Trained music therapist	Once weekly (45 min/session) for 3 weeks	Routine nursing care	HADS
Chen et al., 2016	China	N = 200, Female = 0	35.5(9.75)	Prisoners with mild depression;	Active+receptive music therapy, including music and imagery, improvisation, and song writing	Music therapist	Twice weekly (90 min/session) for 3 weeks	Standard care	BDI
Esfandiari et al., 2014	Iran	N = 30, Female = 30	Not reported	Severe depressive disorder	Music medicine	not reported	90 min/session	Standard care	BDI
Fancourt et al., 2016	UK	N = 45, Female = 37	53.54 (13.85)	Mental health service users	Music medicine	Professional drummer	Once weekly (90 min/session) for 10 weeks	Without any intervention	HADS
Giovagnoli et al., 2017	Italy	N = 39, Female = 24	73.64(7.11)	Mild to moderate Alzheimer’s disease	Active music therapy (Improvisational music therapy)	Music therapist	Twice weekly (45 min/session) for 12 weeks	Cognitive training or neuroeducation	BDI
Harmat et al., 2008	Hungary	N = 94, Female = 73	22.6(2.83)	Seep complaints	Music medicine	Investigators	Once a day (45 min/session) for 3 weeks	listening to an audiobook or no intervention	BDI
Koelsch et al., 2010	Germany	N = 154, Female = 78	24.6	No disease	Active music therapy	Music therapist	Not reported	Individual psychotherapy	POMS
Liao et al., 2018	China	N = 60, Female = 30	61.82(13.20)	Cancer	Receptive music therapy+muscle relaxation training	not reported	Once a day (40 min/session) for 8 weeks	Muscle relaxation training	HADS
Lu et al., 2013	Taiwan China	N = 80, Female = 21	52.02 (7.64)	Schizophrenia	Active music therapy+receptive music therapy	Trained research assistant	Twice weekly (60 min/session) for 5 weeks	Usual care	CDSS
Mahendran et al., 2018	Singapore	N = 68, Female = 56	71.1(5.05)	Mild cognitive impairment	Receptive music therapy	Music therapist	Weekly in the first 3 months, then fortnightly for 6 months.	Standard care without any intervention	GDS-15
Mondanaro et al., 2017	Italy	N = 60, Female = 35	48.20(4.49)	Patients after spine surgery	Active music therapy (improvisational music therapy)	Music therapist	30-minute music therapy session during an 8-hour period within 72 hours after surgery	Standard care without any intervention	HADS
Nwebube et al., 2017	UK	N = 36, Female = 36	Not reported	Pregnant women	Music medicine	Investigators	Once a day (20 min/session) for 12 weeks	Standard care without any intervention	EPDS
Porter et al., 2017	Northern Ireland	N = 184, Female = 73	12.7 (2.5)	Adolescents with behavioural and emotional problems	Active music therapy (improvisational music therapy)	Music therapist	Once weekly (30 min/session) for 13 weeks	Usual care	CES-D
Raglio et al., 2016	Italy	N = 30, Female = 17	64 (10.97)	Amyotrophic lateral sclerosis	Active music therapy	Music therapist	Three times weekly (30 min/session) for 4 weeks	Standard care	HADS
Torres, et al., 2018	Spanish	N = 70, Female = 70	35-65(51.3)	Fibromyalgia	Receptive music therapy	Music therapist	Once weekly (120 min/session) for 12 weeks	Without any additional service	ST/DEP
Wang et al., 2011	China	N = 80, Female = 21	19.35(1.68)	Student	Receptive music therapy	Not reported	Not reported	Without any additional service	SDS
Yap et al., 2017	Singapore	N = 31, Female = 29	74.65(6.4)	Elderly people	Active music therapy (improvisational music therapy)	Experienced instructors	Once weekly (60 min/session) for 11 weeks	Without any intervention	GDS

Note: BDI = Beck Depression Inventory; CDI = Children’s Depression Inventory; CDSS = depression scale for schizophrenia; CES-D = Center for Epidemiologic Studies Depression; CS = Cornell Scale; DMSRIA = Depression Mood Self-Report Inventory for Adolescence; EPDS = Edinburgh Postnatal Depression Scale; GDS-15 = Geriatric Depression Scale-15; GDS-30 = Geriatric Depression Scale-30; HADS = Hospital Anxiety and Depression Scale; HRSD (HAMD) = Hamilton Rating Scale for Depression; MADRS = Montgomery-sberg Depression Rating Scale; PROMIS = Patient Reported Outcomes Measurement Information System; SDS = Self-Rating Depression Scale; State-Trait Depression Questionnaire = ST/DEP; SV-POMS = short version of Profile of Mood States; NA = not available.

**Fig 2:**
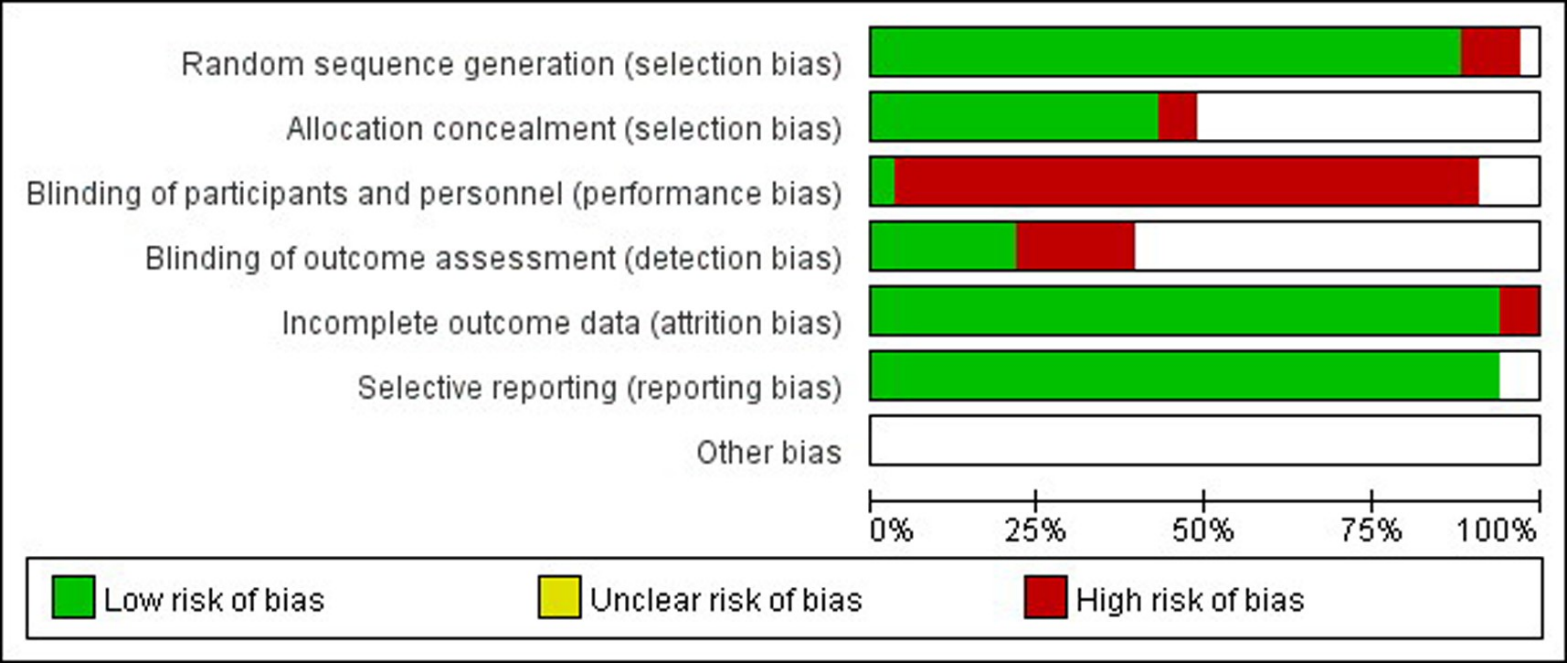
Risk-of-bias graph and risk.

Of the 55 studies, only 2 studies had high risks of selection bias, and almost all of the included studies had high risks of performance bias (Fig 2).

### The overall effects of music therapy

**Fig 3:**
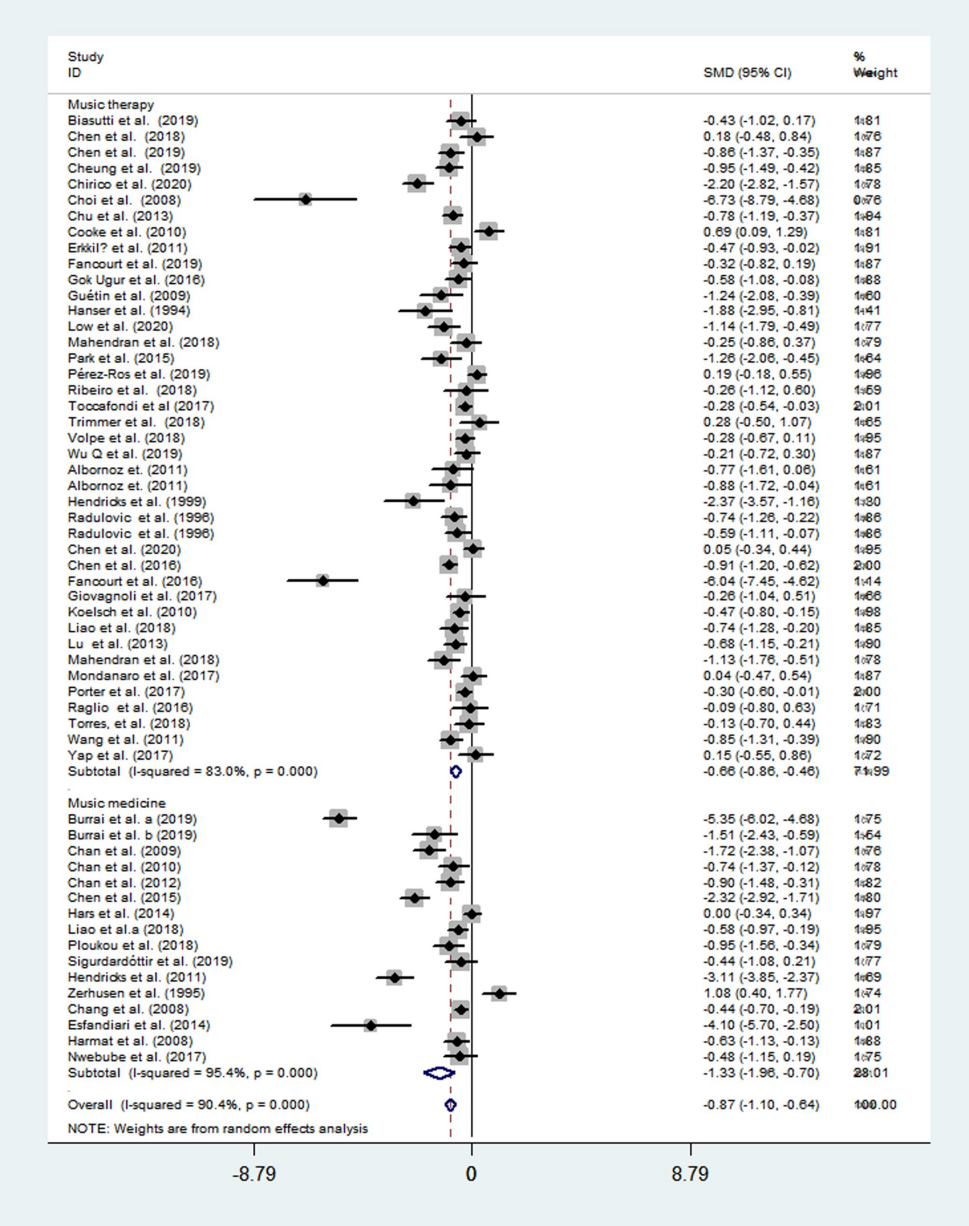
Effects of music therapy and music medicine to reduce depression.

Of the included 55 studies, 39 studies evaluated the music therapy, 17 evaluated the music medicine. Using a random-effects model, music therapy was associated with a significant reduction in depressive symptoms with a moderate-sized mean effect (SMD = −0.66; 95% CI = -0.86 to -0.46; *P*<0.001), with a high heterogeneity across studies (*I*^*2*^ = 83%, *P*<0.001); while, music medicine exhibited a stronger effect in reducing depressive symptom (SMD = −1.33; 95% CI = -1.96 to -0.70; *P*<0.001) (Fig 3).

**Fig 4:**
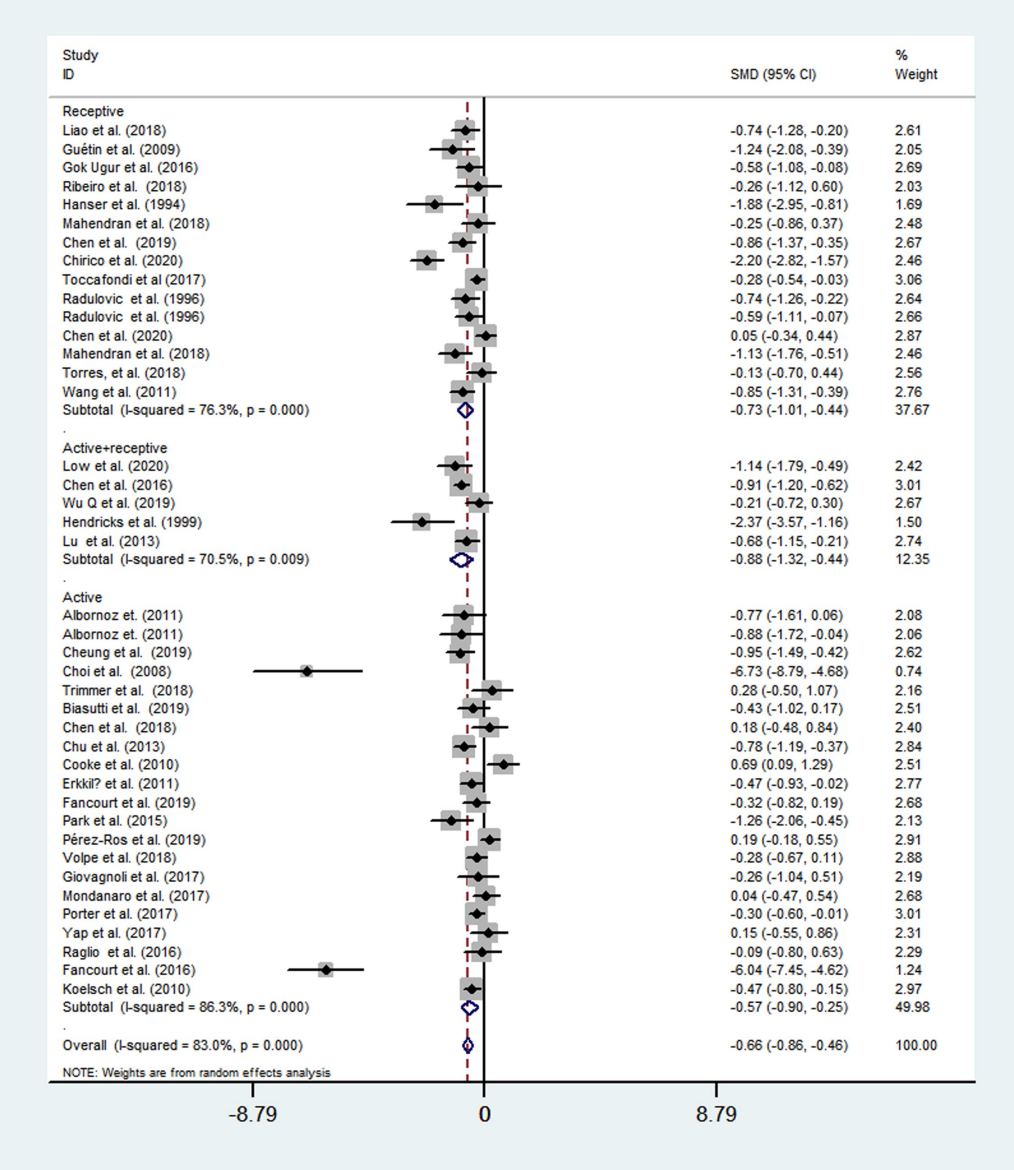
Effects of active music therapy, receptive therapy, and music therapy+receptive therapy to reduce depression.

Twenty studies evaluated the active music therapy using a random-effects model, and a moderate-sized mean effect (SMD = −0.57; 95% CI = -0.90 to -0.25; *P*<0.001) was observed with a high heterogeneity across studies (*I*^*2*^ = 86.3%, *P*<0.001). Fourteen studies evaluated the receptive music therapy using a random-effects model, and a moderate-sized mean effect (SMD = −0.73; 95% CI = -1.01 to -0.44; *P*<0.001) was observed with a high heterogeneity across studies (*I*^*2*^ = 76.3%, *P*<0.001). Five studies evaluated the combined effect of active and receptive music therapy using a random-effects model, and a moderate-sized mean effect (SMD = −0.88; 95% CI = -1.32 to -0.44; *P*<0.001) was observed with a high heterogeneity across studies (*I*^*2*^ = 70.5%, *P*<0.001) (Fig 4).

**Fig 5:**
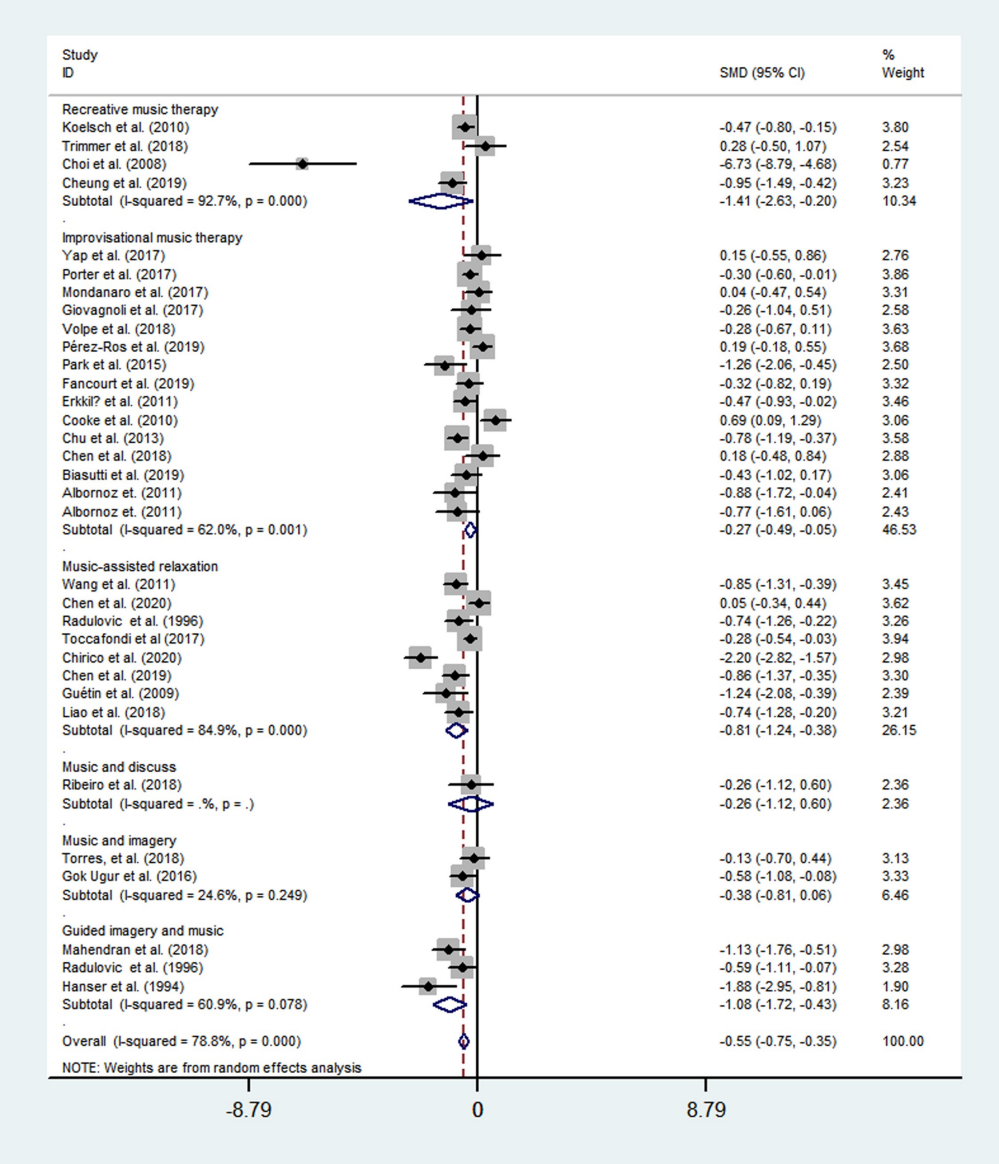
Effects of specific music therapy method to reduce depression.

Among specific music therapy methods, recreative music therapy (SMD = -1.41; 95% CI = -2.63 to -0.20; *P*<0.001), guided imagery and music (SMD = -1.08; 95% CI = -1.72 to -0.43; *P*<0.001), music-assisted relaxation (SMD = -0.81; 95% CI = -1.24 to -0.38; *P*<0.001), music and imagery (SMD = -0.38; 95% CI = -0.81 to 0.06; *P* = 0.312), improvisational music therapy (SMD = -0.27; 95% CI = -0.49 to -0.05; *P* = 0.001), and music and discuss (SMD = -0.26; 95% CI = -1.12 to 0.60; *P* = 0.225) exhibited a different effect respectively (Fig 5).

### Sub-group analyses and meta-regression analyses

We performed sub-group analyses and meta-regression analyses to study the homogeneity. We found that music therapy yielded a superior effect on reducing depression in the studies with a small sample size (20–50), with a mean age of 50–65 years old, with medium intervention frequency (<3 times weekly), with more minutes per session (>60 minutes). We also found that music therapy exhibited a superior effect on reducing depression among people with severe mental disease /psychiatric disorder and depression compared with mental health people. While, whether the country have the music therapy profession, whether the study used group therapy or individual therapy, whether the study was in the outpatients setting or the inpatient setting, and whether the study used a certified music therapist all did not exhibit a remarkable different effect (Table 2). Table 2 also presents the subgroup analysis of music medicine on reducing depression.

**Table 2:** Subgroup analyses of music-based intervention to reduce depression.

Subgroups	Music therapy					Music medicine				
	Trials number	Effects		Heterogeneity		Trials number	Effects		Heterogeneity	
		SMD (95%CI)	*P*	*I*^*2*^*(%)*	*P*		SMD (95%CI)	*P*	*I*^*2*^*(%)*	*P*
Sample size										
20–50	16	-1.24(-2.08, -0.39)	<0.001	143.19	<0.001	7	-1.21(-1.79, -0.62)	<0.001	26.30	<0.001
≥50, <100	17	-0.62(-0.84, -0.38)	<0.001	51.58	<0.001	5	-1.17(-2.45, 0.11)	0.073	86.86	<0.001
≥100	8	-0.36(-0.60, -0.11)	0.005	31.33	<0.001	4	-1.56(-3.10, -0.02)	0.047	206.10	<0.001
Female predominance (>80%)										
Yes	13	-0.73(-1.23, -0.22)	0.005	112.85	<0.001	8	-1.71(-2.76, -0.65)	0.001	247.54	<0.001
No	24	-0.58(-0.81, -036)	<0.001	109.59	<0.001	6	-0.93(-1.32, -0.54)	<0.001	12.51	0.028
Mean age (years)										
<50	20	-0.6(-0.85, -0.35)	<0.001	84.50	<0.001	5	-1.36(-2.30, -0.41)	0.005	69.99	<0.001
50–65	7	-1.43(-2.28, -0.58)	0.001	78.58	<0.001	2	-1.10(-1.66, -0.53)	<0.001	1.22	<0.001
>65	12	-0.48(-0.84, -0.13)	0.008	48.47	<0.001	6	-1.21(-2.66, 0.24)	0.102	237.19	<0.001
Pre-treatment diagnosis										
Mental health	23	-0.58(-0.85, -0.32)	<0.001	141.40	<0.001	10	-1.26(-2.04, -0.47)	0.002	218.03	<0.001
Depression	9	-0.79(-1.13, -0.46)	<0.001	20.83	<0.001	6	-1.49(-2.72, -0.25)	0.018	106.87	<0.001
Severe mental disease /psychiatric disorder	9	-0.78(-1.34, -0.23)	<0.001	62.14	<0.001	0	-	-	-	
Intervention frequency										
Once weekly	21	-0.72 (-1.04, -0.41)	<0.001	118.78	<0.001	7	-1.11(-1.77, -0.44)	0.001	67.58	<0.001
Twice weekly	10	-0.79 (-1.13, -0.46)	<0.001	38.43	<0.001	3	-0.56(-2.49, 1.37)	0.570	53.98	<0.001
≥3 times weekly	6	-0.14 (-0.53, 0.25)	0.476	18.65	0.002	5	-1.67(-3.28, -0.06)	0.042	185.98	<0.001
Time per session (minutes)										
15–40	12	-0.52(-0.86, -0.19)	0.002	59.84	<0.001	9	-1.34(-2.38, -0.29)	0.012	245.42	<0.001
41–60	10	-0.56(-0.99, -0.13)	0.012	62.25	<0.001	6	-0.96(-1.65, -0.27)	0.006	57.46	<0.001
>60	12	-0.96(-1.46, -0.47)	<0.001	81.18	<0.001	1	-4.1(-5.7, -2.50)	<0.001	0	-
Country having music therapy profession										
Yes	39	-0.65(-0.86, -0.45)	<0.001	234.06	<0.001	13	-1.26(-1.99, -0.53)	0.001	309.93	<0.001
No	2	-0.83(-1.42, -0.23)	<0.001	0.03	0.864	3	-1.60(-2.86, -0.34)_	0.003	16.49	<0.001
Group therapy or individual therapy										
Group therapy	30	-0.66 (-0.92, -0.41)	<0.001	177.02	<0.001	8	-1.23(-2.10, -0.36)	0.006	128.59	<0.001
Individual therapy	10	-0.67 (-1.05, -0.29)	0.001	56.14	<0.001	7	-1.57(-2.71, -0.42)	0.007	190.82	<0.001
Setting										
Outpatient	16	-0.89(-1.30, -0.47)	<0.001	103.66	<0.001	12	-1.26(-1.94, -0.57)	<0.001	255.53	<0.001
Inpatient	22	-0.57(-0.83, -0.31)	<0.001	127.51	<0.001	3	-0.91(-3.10, 1.28)	0.414	54.87	<0.001
Used a certified music therapist										
Yes	32	-0.69 (-0.88, -0.49)	<0.001	131.76	<0.001	-	-	-	-	-
No	5	-0.93 (-2.12, 0.25)	0.123	82.69	<0.001	10	-1.71(-2.61, -0.81)	<0.001	234.94	<0.001

**Fig 6:**
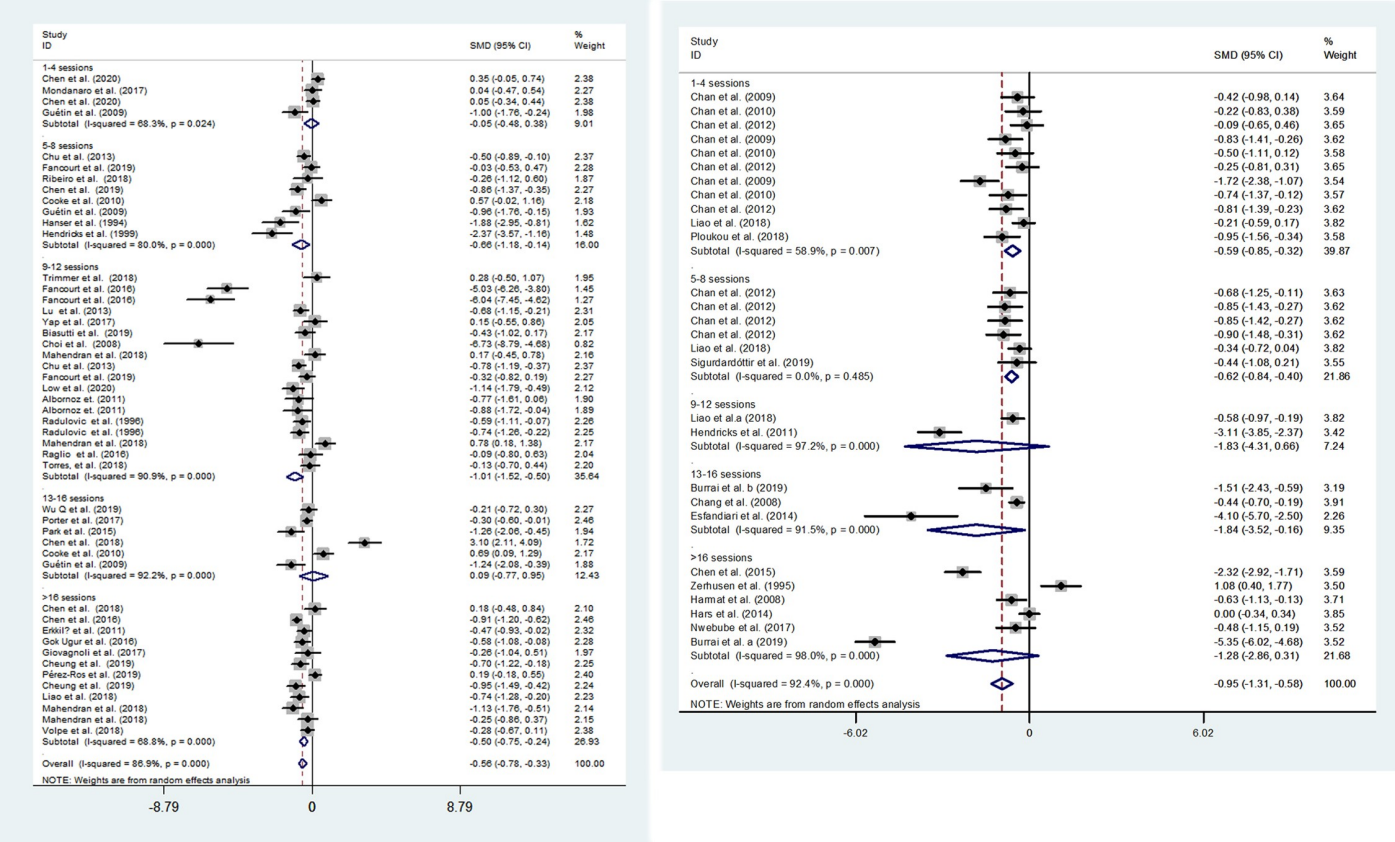
Effects of music therapy and music medicine to reduce depression by total sessions. A, evaluating the effect of music therapy; B, evaluating the effect of music medicine.

In the subgroup analysis by total session, music therapy and music medicine both exhibited a stronger effects of short (1–4 sessions) and medium length (5–12 sessions) compared with long intervention periods (>13sessions) (Fig 6). Meta-regression demonstrated that total music intervention session was significantly associated with the homogeneity between studies (*P* = 0.004) (Table 3).

**Table 3:** Meta-regression analysis of the main characteristics of the 33 studies.

Characteristics	Music therapy		Music medicine	
	Coef. 95%CI	*P*	Coef. 95%CI	*P*
Sample size	0(-0.01, 0.03)	0.704	0(-0.01, 0.01)	0.926
Mean age (years)	0.01(-0.03, 0.05)	0.39	-	-
Setting				
Inpatient	1		1	
Outpatient	0.13(-1.98, 2.23)	0.901	1.48(-0.59, 3.55)	0.139
Pre-treatment diagnosis				
Mental health	1	1	1	
Depression	-0.24(-1.20, 0.72)	0.622	-0.24(-2.08, 1.61)	0.789
Severe mental disease /psychiatric disorder	-0.22(-1.18, 0.75)	0.652	-	
Music therapy method				
Active music therapy	1			
Receptive music therapy	0.13(-1.89, 2.14)	0.895	-	-
Active+receptive	0.48(-2.26, 3.21)	0.716	-	-
Total music intervention sessions	0.01(-0.05, 0.06)	0.83	-0.02(-0.03, -0.01)	0.004
Music intervention frequency	-0.08(-1.74, 1.58)	0.918	0.45(-0.66, 1.57)	0.376
Time per session (minutes)	-0.01(-0.04, 0.02)	0.482	-0.01(-0.07, 0.05)	0.778

### Sensitivity analyses

We performed sensitivity analyses and found that re-estimating the pooled effects using fixed effect model, using trim and fill analysis, excluding the paper without information regarding music therapy, excluding the papers with more high biases, excluding the papers with small sample size (20< n<30), excluding the studies focused on the people with a severe mental disease, and excluding the papers using an infrequently used scale yielded the similar results, which indicated that the primary results was robust (Table 4).

**Table 4:** Sensitivity analyses of the main outcomes [SMD (95%CI)].

Outcomes	Trials number	Effects		Heterogeneity		Egger’s est	
		SMD (95%CI)	*P*	*I*^*2*^*(%)*	*P*	*a*	*P*
Music therapy							
Using fixed effect model	41	-0.50 (-0.58, -0.43)	<0.001	83	<0.001	-2.82(-4.71, -0.93)	0.005
Using trim and fill analysis	41	-0.66 (-0.86, -0.46)	<0.001	-	<0.001	-	-
Excluding the paper without information regarding music therapy (Chirico et al., 2020; Koelsch et al., 2010; Toccafondi et al., 2017; Porter et al., 2017)	37	-0.66 (-0.88, -0.43)	<0.001	82.2	<0.001	-3.03(-5.26, -0.81)	0.009
Excluding the papers with high bias (Toccafondi et al., 2017 and Fancourt et al., 2019)	39	-0.69 (-0.91, -0.47)	<0.001	83.6	<0.001	-2.95(-5.04, -0.86)	0.007
Excluding the papers with small sample size (20< n<30)	35	-0.57 (-0.77, -0.38)	<0.001	81.3	<0.001	2.22(-4.53, 0.08)	0.058
Excluding the studies focused on the people with a severe mental disease (Choi et al., 2008; Cheung et al. 2019)	32	-0.64(-0.86, -0.42)	<0.001	82.1	<0.001	‘-2.54(-4.67, -0.40)	0.022
Excluding the papers using an infrequently used scale (Erkkilä et al., 2011; Chen et al., 2015; Cheung et al., 2019; Chirico et al., 2020; Park et al., 2015; Sigurdardóttir et al., 2019; Wu et al., 2019; Low et al., 2020)	34	-0.62 (-0.84, -0.39)	<0.001	83.2	<0.001	-2.63(-4.67, -0.60)	0.013
Music medicine							
Using fixed effect model	16	-0.86(-0.98, -0.73)	<0.001	95.4	<0.001	-5.78(-11.65, 0.10)	0.053
Using trim and fill analysis	16	-1.33(-1.96, -0.70)	<0.001	-	<0.001	-	-
Excluding the papers with small sample size (20< n<30) [49]	15	-1.32(-1.98, -0.66)	<0.001	95.7	<0.001	-6.09(-12.53, 0.36)	0.062
Excluding the papers using an infrequently used scale (Chen et al., 2015)	14	-1.25(-1.92, -0.57)	<0.001	95.7	<0.001	-5.71(-12.38, 0.98)	0.98

### Evaluation of publication bias

**Fig 7:**
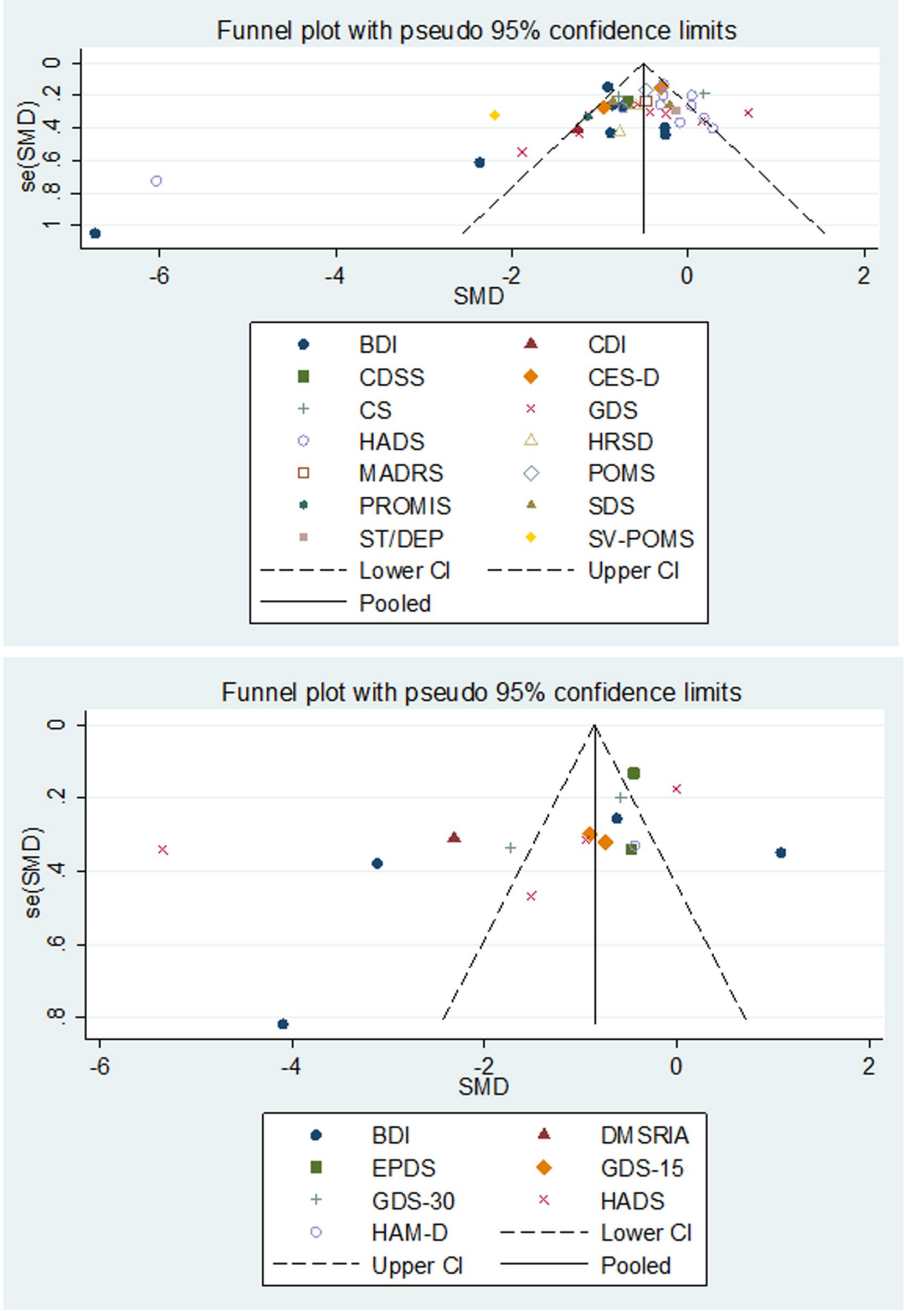
Funnel plot illustrating proneness to publication bias for the included studies. A, evaluating the publication bias of music therapy; B, evaluating the publication bias of music medicine; BDI = Beck Depression Inventory; CDI = Children’s Depression Inventory; CDSS = depression scale for schizophrenia; CES-D = Center for Epidemiologic Studies Depression; CS = Cornell Scale; DMSRIA = Depression Mood Self-Report Inventory for Adolescence; EPDS = Edinburgh Postnatal Depression Scale; GDS-15 = Geriatric Depression Scale-15; GDS-30 = Geriatric Depression Scale-30; HADS = Hospital Anxiety and Depression Scale; HRSD (HAMD) = Hamilton Rating Scale for Depression; MADRS = Montgomery-sberg Depression Rating Scale; PROMIS = Patient Reported Outcomes Measurement Information System; SDS = Self-Rating Depression Scale; State-Trait Depression Questionnaire = ST/DEP; SV-POMS = short version of Profile of Mood Stat.

We assessed publication bias using Egger’s linear regression test and funnel plot, and the results are presented in Fig 7. For the main result, the observed asymmetry indicated that either the absence of papers with negative results or publication bias.

## Discussion

Our present meta-analysis exhibited a different effect of music therapy and music medicine on reducing depression. Different music therapy methods also exhibited a different effect, and the recreative music therapy and guided imagery and music yielded a superior effect on reducing depression compared with other music therapy methods. Furthermore, music therapy and music medicine both exhibited a stronger effects of short and medium length compared with long intervention periods. The strength of this meta-analysis was the stable and high-quality result. Firstly, the sensitivity analyses performed in this meta-analysis yielded similar results, which indicated that the primary results were robust. Secondly, considering the insufficient statistical power of small sample size, we excluded studies with a very small sample size (n<20).

Some prior reviews have evaluated the effects of music therapy for reducing depression. These reviews found a significant effectiveness of music therapy on reducing depression among older adults with depressive symptoms, people with dementia, puerpera, and people with cancers [4, 5, 73–76]. However, these reviews did not differentiate music therapy from music medicine. Another paper reviewed the effectiveness of music interventions in treating depression. The authors included 26 studies and found a signifiant reduction in depression in the music intervention group compared with the control group. The authors made a clear distinction on the definition of music therapy and music medicine; however, they did not include all relevant data from the most recent trials and did not conduct a meta-analysis [77]. A recent meta-analysis compared the effects of music therapy and music medicine for reducing depression in people with cancer with seven RCTs; the authors found a moderately strong, positive impact of music intervention on depression, but found no difference between music therapy and music medicine [78]. However, our present meta-analysis exhibited a different effect of music therapy and music medicine on reducing depression, and the music medicine yielded a superior effect on reducing depression compared with music therapy. The different effect of music therapy and music medicine might be explained by the different participators, and nine studies used music therapy to reduce the depression among people with severe mental disease /psychiatric disorder, while no study used music medicine. Furthermore, the studies evaluating music therapy used more clinical diagnostic scale for depressive symptoms.

A meta-analysis by Li et al. [74] suggested that medium-term music therapy (6–12 weeks) was significantly associated with improved depression in people with dementia, but not short-term music therapy (3 or 4 weeks). On the contrary, our present meta-analysis found a stronger effect of short-term (1–4 weeks) and medium-term (5–12 weeks) music therapy on reducing depression compared with long-term (≥13 weeks) music therapy. Consistent with the prior meta-analysis by Li et al., no significant effect on depression was observed for the follow-up of one or three months after music therapy was completed in our present meta-analysis. Only five studies analyzed the therapeutic effect for the follow-up periods after music therapy intervention therapy was completed, and the rather limited sample size may have resulted in this insignificant difference. Therefore, whether the therapeutic effect was maintained in reducing depression when music therapy was discontinued should be explored in further studies. In our present meta-analysis, meta-regression results demonstrated that no variables (including period, frequency, method, populations, and so on) were significantly associated with the effect of music therapy. Because meta-regression does not provide sufficient statistical power to detect small associations, the non-significant results do not completely exclude the potential effects of the analyzed variables. Therefore, meta-regression results should be interpreted with caution.

Our meta-analysis has limitations. First, the included studies rarely used masked methodology due to the nature of music therapy, therefore the performance bias and the detection bias was common in music intervention study. Second, a total of 13 different scales were used to evaluate the depression level of the participators, which may account for the high heterogeneity among the trials. Third, more than half of those included studies had small sample sizes (<50), therefore the result should be explicated with caution.

## Conclusion

Our present meta-analysis of 55 RCTs revealed a different effect of music therapy and music medicine, and different music therapy methods also exhibited a different effect. The results of subgroup analyses revealed that the characters of music therapy were associated with the therapeutic effect, for example specific music therapy methods, short and medium-term therapy, and therapy with more time per session may yield stronger therapeutic effect. Therefore, our present meta-analysis could provide suggestion for clinicians and policymakers to design therapeutic schedule of appropriate lengths to reduce depression.

## Supporting information

S1 Checklist PRISMA checklist. (DOC)

S1 Dataset (XLSX)
